# Importation of Poliomyelitis by Travelers

**DOI:** 10.3201/eid1402.071245

**Published:** 2008-02

**Authors:** Annelies Wilder-Smith, Karin Leder, Paul A. Tambyah

**Affiliations:** * National University Singapore, Singapore; † Monash University, Melbourne, Victoria, Australia

**Keywords:** Poliomyelitis, importation of polio, international travelers, letter

## Abstract

Importation of Poliomyelitis by Travelers

## To the Editor 

In July 2007, an Australian traveler imported polio from Pakistan to Australia (*1*). He was a 22-year-old man who had immigrated to Australia and had traveled to his country of origin (Pakistan) to visit friends and relatives. Pakistan is one of 4 countries (Afghanistan, India, Nigeria, Pakistan) where polio is still endemic. A diagnosis of polio was made shortly after his return to Australia. Australia was certified as polio-free in 2000. Australia will not be the last industrialized country affected by importation of polio. All countries are at risk until polio has been completely eradicated.

Between 2003 and 2006, polio was imported by travelers (e.g., refugees, pilgrims, traders) to 24 polio-free countries (*2*). The origin of these importations was largely the 4 countries where polio transmission was never completely interrupted. The importations resulted in about 1,400 secondary cases (*2*). The resurgence of polio by international spread was a setback to the Global Polio Eradication Initiative that had successfully decreased the number of polio-affected countries to only 9 in 2002.

The revised International Health Regulations, IHR (2005) (*3*), entered into legal force on June 15, 2007. These regulations provide the legal framework for coordination of the international effort to reduce or prevent international spread of diseases of public health concern. IHR (2005) (*2*) lists polio as one of the diseases of public health emergencies of international concern. Preventing importation of polio into polio-free countries is therefore a test case for the revised International Health Regulations (*4*). Compared to the previous IHR (1969), IHR (2005) has moved away from the definition of fixed maximum measures relating to specific diseases and instead focuses on the issuance of context-specific recommendations, made either on a temporary emergency basis (a temporary recommendation) or routinely for established ongoing risks of disease spread (a standing recommendation).

One strategy to protect polio-free countries from reintroduction of wild poliovirus is by requiring proof of polio vaccination for all incoming travelers from polio-endemic countries. This was proposed by the Advisory Committee on Poliomyelitis Eradication in October 2006. The rationale is similar to that used for yellow fever, currently the only disease for which proof of vaccination may be required for travelers as a condition of entry to a country. The proposal of the Advisory Committee of Poliomyelitis Eradication was discussed at the World Health Assembly in May 2007 (*5*). Although the main strategy for polio eradication continues to be attaining high vaccination coverage against polio in all countries, the 193 member states have also adopted the resolution to “continue to examine and disseminate measures that member states can take for reducing the risk and consequences of international spread of polioviruses, including, if and when needed, the consideration of Temporary or Standing Recommendations, under the International Health Regulations (2005)” (*3*).

The recent polio importation by an inadequately vaccinated traveler would add impetus to such considerations. However, this case also shows that focusing on travelers from polio-endemic countries alone may not be sufficient. Immigrants from developing countries to industrialized countries who subsequently return to their home countries to visit friends and relatives may also be at increased risk if traveling to polio-endemic countries, in particular as many may not have received adequate childhood vaccination including vaccination against polio (*6*). Targeting those visiting friends and relatives is therefore a potential additional strategy to reduce the risk for the worldwide spread of polio.
