# Supplementary material for: Importation of Poliomyelitis by Travelers
Source: Emerg Infect Dis. 2008 Feb;14(2):351–2. doi: 10.3201/eid1402.071245 (PMC2600199; doi:10.3201/eid1402.071245)
Supplement: Related texts

# Reply: In Response

**Authors:** Harry F. Hull

**Affiliations:** * HF Hull and Associates, LLC, St. Paul, Minnesota, USA

After poliomyelitis was imported into Australia, Wilder-Smith and colleagues (*1*) call for proof of vaccination for travelers from polio-endemic countries. Although superficially attractive, their recommendation won’t be extremely effective, will be burdensome for polio-endemic and polio-free countries, and is unnecessary.

Documenting vaccination may slightly reduce, but will not eliminate, importations. Vaccination simply does not provide high-level protection against poliovirus infection. Children recently vaccinated with either oral poliomyelitis virus (OPV) or inactivated poliomyelitis virus (IPV) shed poliovirus following a challenge OPV dose (*2*). Because secretory immunity falls rapidly, a high percentage of persons vaccinated years or even decades ago will become transiently infected when exposed to poliovirus and will excrete virus for weeks. Lower vaccine efficacy in developing countries (*3*) further compounds the issue.

Screening programs are likely to be costly and will not be simple to implement. Unanswered questions include the following: Is a single dose of IPV or OPV immediately before departure adequate? Are boosters needed? Why not include countries with imported wild or vaccine-derived poliovirus (VDPV) outbreaks? Can polio-free areas of polio-endemic countries (e.g., Kerala) be exempted? Must records be certified? Can fraudulent vaccinations be detected or prevented?

Data on importations clarifies any need for requiring vaccination of travelers entering polio-free countries. Polioviruses are imported regularly, yet outbreaks are rare. The Australian case comprised 1 imported case. Similarly, no paralytic cases followed the recent importation of a poliovirus from Chad into Switzerland (*4*) or the 2005 Minnesota VDPV infections (*5*). The United Kingdom has been polio-free for decades despite close ties with India, Pakistan, and Nigeria. Polio outbreaks (both wild and VDPV) occur where immunization coverage is low. The last major outbreak in Western Europe occurred in a Dutch religious group that refuses immunization. The 2005–06 global outbreak affected polio-free countries where polio immunization coverage had fallen after transmission was interrupted.

Countries at risk for polio importation because of low vaccination coverage should focus on improving their immunization programs, not vaccinating and screening travelers. Australia and other polio-free countries can best protect themselves against importations by supporting eradication efforts in polio-endemic countries.

## References

[1] Wilder-Smith A, Leder K, Paul T. Importation of poliomyelitis by travelers. Emerg Infect Dis. 2008;14:351–2.1825814510.3201/eid1402.071245PMC2600199

[2] Onorato IM, Modlin JF, McBean AM, Thomas ML, Losonsky GA, Bernier RH, Mucosal immunity induced by enhanced-potency inactivated and oral polio vaccines. J Infect Dis. 1991;163:1–6.184580610.1093/infdis/163.1.1

[3] WHO Collaborative Study Group on Oral and Inactivated Poliovirus Vaccines. Combined immunization of infants with oral and inactivated poliovirus vaccines: results of a randomized trial in The Gambia, Oman, and Thailand. Bull World Health Organ. 1996;74:253–68.8789924PMC2486925

[4] Global Polio Eradication Initiative. Wild poliovirus isolated in Switzerland’s sewer system; insignificant risk of outbreak; 2007 Nov 12 [cited 26 Nov 2007]. Available from http://www.polioeradication.org/content/general/LatestNews200711.asp

[5] Poliovirus infections in four unvaccinated children—Minnesota, August,-October 2005. MMWR Morb Mortal Wkly Rep. 2005;54:1053–5.Centers for Disease Control and Prevention.16237378
